# Risk factors for rectal colonization of carbapenem-resistant Enterobacteriaceae in a tertiary care hospital: a case-control study from Turkey

**DOI:** 10.3906/sag-1810-65

**Published:** 2019-02-11

**Authors:** Fatma ESER, Gül Ruhsar YILMAZ, Rahmet GÜNER, İmran HASANOĞLU, Fatma Yekta ÜRKMEZ KORKMAZ, Ziya Cibali AÇIKGÖZ, Mehmet Akın TAŞYARAN

**Affiliations:** 1 Department of Infectious Disease and Clinical Microbiology, Ankara Atatürk Training and Research Hospital,Yıldırım Beyazıt University, Ankara Turkey; 2 Department of Infectious Disease and Clinical Microbiology, Faculty of Medicine, Yıldırım Beyazıt University, Ankara Turkey; 3 Department of Clinical Microbiology, Faculty of Medicine, Yıldırım Beyazıt University, Ankara Turkey

**Keywords:** Enterobacteriaceae, colonization, risk factor, *Klebsiella pneumoniae*, carbapenem

## Abstract

**Background/aim:**

This study aimed to evaluate the risk factors of patients colonized with carbapenem-resistant Enterobacteriaceae (CRE).

**Materials and methods:**

The study was conducted between January 2010 and March 2016. The colonized group consisted of patients who had a CRE strain in their rectal swab cultures, whereas patients with negative rectal surveillance cultures for CRE who were concurrently hospitalized in the same units with the colonized group patients were included in the control group.

**Results:**

The number of patients in the colonized and the control group was 71 and 120, respectively. Both groups were evaluated for demographic and healthcare-associated characteristics. Isolated microorganisms in rectal surveillance cultures for CRE were *Klebsiella pneumoniae* (75.5%), *Escherichia coli* (15.5%), *Enterobacter cloacae* (4.2%), *Klebsiella oxytoca* (1.4%), and *Klebsiella terrigena* (1.4%). The isolates were resistant to imipenem, meropenem, and ertapenem (52.1%, 73.2%, and 100%, respectively). In multivariate analysis, presence of decubitus, colistin usage, glycopeptide usage, and fluoroquinolone usage were found to be independent risk factors for CRE colonization. There was no significant difference between the two groups with regards to mortality (P = 0.070).

**Conclusion:**

These results are in agreement with the current literature. The findings of this study could be useful for improvement of infection control strategies related to CRE.

## 1. Introduction

Infections caused by carbapenem-resistant Enterobacteriaceae (CRE) occur very frequently. High mortality rates among these infections are a serious threat for patients, especially in long-term care facilities, as reported by Sanchez et al. and Şenbayrak et al. (1,2). The Centers for Disease Control and Prevention recommends screening for CRE either via point prevalence surveys or active surveillance cultures based on the facility’s characteristics. Determining the frequency of CRE infections and colonizations is critical for management of nosocomial infections (3). Appropriate interventions are associated with significant reductions in CRE colonization and CRE infections in long-term acute-care hospitals (4). 

During the last decade, prior antibiotic exposure, comorbidity, immunosuppression, and indwelling catheter usage were identified as risk factors for rectal colonization with CRE (5,6). Although risk factors for acquisition of rectal colonization in Turkey were reported for pediatric and neonatal intensive care units (7), for adult patient populations the risk factors for acquisition of rectal CRE colonization in Turkey have not been reported. 

The aim of this study is to evaluate the clinical characteristics and risk factors for patients with acquired CRE rectal colonization. 

## 2. Materials and methods

### 2.1. Study design and population

The study was conducted at a tertiary education and research hospital, from January 2010 to March 2016. The data were retrospectively collected from the hospital’s infection control committee records. Before November 2013 the surveillance program was carried out as a point prevalence survey in selected units of the hospital tracking individual clinical infections caused by CRE if they were identified. However, after November 2013, the hospital adopted an active surveillance protocol by obtaining rectal swab cultures for CRE on admission to the intensive care unit (ICU) and routinely once a week from all patients in the ICUs. Furthermore, in selected units, after positive detection of a clinical infection caused by CRE, rectal swabs were collected weekly. The patients in the internal medicine ICU, general surgery ICU, transplantation ICU, anesthesiology ICU, hematology-oncology, and chest diseases services were included in the study. 

Patients who had a CRE strain in their rectal swab cultures were included in the colonized group. Patients with negative rectal surveillance cultures for CRE who were concurrently hospitalized in the same units with the colonized group patients were included in the control group.

All patients’ data were obtained from the hospital database and infection control committee records. Demographic features, Acute Physiology and Chronic Health Evaluation (APACHE II), Simplified Acute Physiology Score II (SAPS II), Glasgow Coma Scale (GCS) score, and Charlson Comorbidity Score at the time of admission to the hospital were evaluated. Diabetes mellitus, chronic renal failure, length of hospitalization, hospitalization within the last 6 months, antibiotic use within the last 3 months and the last 1 year, admission to the ICU, the presence of a central venous catheter, urinary catheter, decubitus ulcer, colostomy, surgery, and invasive procedures were evaluated as potential risk factors. Tracheostomy, percutaneous endoscopic gastrostomy, endoscopy, endoscopic retrograde cholangiopancreatography, coronary angiography, bronchoscopy, nephrostomy, and chest tube insertion were identified as invasive procedures. Only antibiotic usages of 3 days or more were considered. In addition, CRE strains and susceptibility tests for carbapenems were evaluated for the colonized group.

### 2.2. Microbiology 

Rectal specimens were cultured simultaneously on Muller-Hilton agar and eosin methylene blue (EMB) agar media containing 2 mg/L ertapenem and incubated overnight at 35 °C. Single-colony culturing on EMB medium was carried out for bacterial identification. Conventional methods and when needed the API 20 NE (BioMérieux) identification system were used for species-level identification of colonies. The carbapenem resistance was also confirmed by E tests (BioMérieux). Carbapenem resistance for Enterobacteriaceae is defined as resistance to either imipenem, ertapenem, or meropenem (minimum inhibitory concentrations of ≥4 µg/mL for meropenem, ≥4 µg/mL imipenem, and ≥2 µg/mL for ertapenem) (3,8).

### 2.3. Statistical analysis

In univariate analyses, the chi-square test was used for categorical variables and Student’s t-test was used for continuous variables. Significant variables according to the chi-square and Student’s t-tests were used to build a model in logistic regression analyses. All statistical evaluations were performed with a 5% type-I error margin. Therefore, statistical significance was assigned at P < 0.05. SPSS 21 (IBM Inc., Armonk, NY, USA) was used for data analysis.

This study was conducted with the local ethical committee approval of our hospital. 

## 3. Results

Seventy-one patients with CRE in rectal swab cultures were included in the study as the colonized group. One hundred and twenty patients who met the criteria defined in Section 2 were included as the control group. Before the active surveillance program, only one patient was detected with rectal swab culture positivity for CRE. 

Mean ages for the colonized and control groups were 65.8 and 62.8 years, respectively. Male patients accounted for 50.7% of the colonized and 52.5% of the control group. There were no significant differences between the colonized and control groups in terms of age (P = 0.323), sex (P = 0.924), and clinical scores (Table 1).

**Table 1:** Demographic characteristics and clinical scores.

	Colonized group	Control group	P
	Mean ± SD	Mean ± SD	
Age	65.8 ± 17.7	62.8 ± 18.8	0.323
GCS	10.8 ± 4	11.6 ± 3.6	0.216
APACHE II	15.1 ± 7.4	13.3 ± 7.7	0.141
Charlson score	4.3 ± 3.5	4.4 ± 2.5	0.227
SAPS II	37.8 ± 15.7	36.5 ± 16.4	0.731

GCS: Glasgow Coma Scale, APACHE: Acute Physiology and Chronic Health Evaluation, SAPS: Simplified Acute Physiology Score.

Of the colonized group 95.8% of patients and of the control group 85.8% of patients were followed up in ICUs (P = 0.071). Most of the patients of study population were followed in the anesthesiology ICU. The most common diagnoses on admission to the hospital were respiratory tract diseases (29.6%), emergency surgery/trauma (21.1%), and neurological diseases (15.5%) in the CRE-colonized group.

Clinical characteristics of the colonized and control groups are given in Table 2. In univariate analysis, rates of admission to the ICU (P = 0.026), invasive procedure history (P = 0.043), presence of decubitus ulcer (P = 0.008), immobilization (P = 0.036), and antibiotic use within 3 months (P < 0.001) were higher in the colonized patient group when compared to the control group patients. When antibiotic use in the previous 3 months was evaluated with regards to specific antibiotic groups, penicillins (P = 0.001), polymyxin (P < 0.001), glycopeptides (P = 0.006), carbapenems (P < 0.001), fluoroquinolones (P = 0.012), and linezolid (P = 0.022) usage was found to be higher in the colonized group (Table 2). 

**Table 2:** Clinical characteristics of colonized and control groups (univariate analysis).

Clinical characteristics	Colonized group(n = 71) n (%)	Control group(n = 120) n (%)	P
Diabetes mellitus	14 (19.7)	33 (27.5)	0.198
Chronic renal disease	6 (8.5)	17 (14.2)	0.221
Immunosuppression	14 (19.7)	18 (15)	0.439
Admission to ICU	66 (93)	98 (81.7)	0.026
Cardiovascular disease	43 (60.6)	58 (48.4)	0.063
Central venous catheter	58 (81.7)	86 (71.7)	0.102
Urinary catheter	65 (91.5)	98 (81.7)	0.055
Surgery	26 (36.6)	49 (40.8)	0.487
Invasive procedure*	46 (63.4)	58 (48.4)	0.043
Pulmonary disease	21 (29.6)	25 (20.8)	0.174
Decubitus	35 (49.3)	35 (29.2)	0.008
Hospitalization within the last 6 months	46 (64.8)	83 (69.2)	0.402
Mechanical ventilation	54 (76.1)	79 (65.8)	0.114
Colostomy	6 (8.5)	6 (5)	0.373
Gastrostomy	18 (25.4)	26 (21.7)	0.616
Immobilization	61 (85.9)	85 (70.8)	0.036
Antibiotic use in previous 3 months	71 (100)	99 (82.5)	<0.001
Penicillins	45 (63.4)	47 (39.2)	0.001
Polymyxin	38 (53.5)	22 (18.3)	<0.001
Glycopeptides	30 (42.3)	28 (23.3)	0.006
Carbapenems	48 (67.6)	49 (40.8)	<0.001
Cephalosporins	40 (56.3)	62 (51.7)	0.532
Linezolid	14 (19.7)	10 (8.3)	0.022
Daptomycin	6 (8.5)	9 (7.5)	0.813
Fluoroquinolones	14 (19.7)	9 (7.5)	0.012
Aminoglycosides	5 (7)	6 (5)	0.541
Antibiotic use in previous 1 year	7 (9.9)	8 (6.7)	0.428

ICU: Intensive care unit. *Tracheostomy, endoscopy, thoracic tube, bronchoscopy, coronary angiography, nephrostomy, endoscopic retrograde cholangiopancreatography.

Isolated microorganisms in rectal surveillance cultures for CRE were *Klebsiella pneumoniae* (75.5%), *Escherichia coli* (15.5%), *Enterobacter cloacae* (4.2%), *Klebsiella oxytoca* (1.4%), and *Klebsiella terrigena* (1.4%). Carbapenem susceptibility of the CRE isolates is given in Table 3.

**Table 3:** Carbapenem susceptibilities of CRE isolates.

Carbapenems	Sensitive n (%)	Intermediate n (%)	Resistant(%)
Imipenem	26 (36.6)	8 (11.3)	37 (52.1)
Meropenem	16 (22.5)	2 (2.8)	52 (73.2)
Ertapenem			71 (100)

In logistic regression analysis, independent predictors of rectal CRE colonization were the presence of decubitus ulcer, polymyxin, glycopeptides, and fluoroquinolone usage (Table 4).

**Table 4:** Risk factors for CRE colonization (multivariate analysis).

	OR	%95 CI	P
Decubitus	2.387	1.284–4.438	0.006
Polymyxin use	3.393	1.503–7.657	0.003
Glycopeptide use	2.643	1.24–5.634	0.012
Fluoroquinolone use	3.387	1.244–9.22	0.017

The hospital mortality rates were 40% in the control group and 53% in the colonized group (P = 0.070). Mean length of stay (LOS) in the patients before acquiring CRE colonization was 38.4 ± 42.6 days. In the control group, the mean LOS was 29.4 ± 32.5 days at the time the patient was included in the control group with a negative rectal swab culture for CRE (P = 0.055). In addition, the total LOS was higher in the colonized group than in the control group (59.3 ± 45.4 and 46.4 ± 46.1 days, resprectively, P = 0.001).

## 4. Discussion

Evaluating the risk factors associated with CRE colonization is crucial for identifying high-risk patients and could be useful for implementing effective strategies for management of CRE infection/colonization control (9). It is reported that CRE colonization can lead to infections that are difficult to treat. Asymptomatic colonized patients are reservoirs for spreading multidrug-resistant microorganisms (10,11)*. *The rectal colonization rate of CRE infected patients is about 80% in Turkey (12). For patients in ICUs, the risk of infection with CRE is associated with twofold increased risk of infection with the colonized strain (13)*. *CRE reduction strategies and targets are recommended to be added to infection control programs for healthcare facilities (3,14)*.*

In our hospital, a point prevalence survey was performed in 2010 after detection of a patient with a urinary tract infection caused by CRE. Before November 2013, surveillance for CRE was conducted via point prevalence surveys when an infection occurred with CRE. Afterwards, active surveillance was adopted in the ICUs and in selected units where CRE infection was detected. Most of the patients (98.6%) were detected with the active surveillance program. A decrease in the number of CRE-colonized patients was observed in the second year of the active surveillance program. 

In accordance with previous studies, we found no difference between the control group and CRE-colonized patients in terms of age and sex (9,13)*. *Several studies reported that poor patient status is a risk factor for the development of resistant bacterial infection and mortality (10,15)*. *However, there are only a few studies evaluating APACHE II and Charlson scores in terms of risk factors for colonized patients with CRE. Although Dickstein et al. reported high APACHE II scores in both colonized and infected groups, APACHE II scores were not associated with increased risk of CRE colonization and infection in their study (13). Bhargava et al. concluded that high Charlson score was a risk factor for CRE colonization (6)*. *In our hospital, patients who were infected with CRE had higher SAPS II scores compared with the control group. However, GCS, APACHE II, and Charlson scores were similar in the two groups (16). The clinical scores indicating poor patient condition were similar in the colonized and the control group.

Studies evaluating risk factors for CRE colonization or carriage reported that carbapenem usage, history of infection with resistant bacteria within the last 6 months, admission to the ICU, hematological malignancy, invasive procedures (17), immunosuppression, presence of indwelling devices, and antibiotic use in the last 3 months (6) were found to be higher in CRE-colonized groups. In multivariate analysis, carbapenem use, hematological malignancy, intensive care unit admission, invasive procedures (17), foreign body presence (18), Charlson score elevation, immunosuppression, and invasive instrument use (6) were reported as risk factors. In addition, Torres-Gonzales et al. and Schwaber et al. reported that admission to the ICU is a risk factor for CRE colonization and CRE infection (17,19). In our study, 95.7% of CRE-colonized patients were in ICUs. Therefore, we did not evaluate if hospitalization in the ICU was a risk factor.

Schechner et al. revealed that decubitus ulcer is not associated with rectal carriage of CRE (20). Recommendations for CRE management in healthcare facilities involve chlorhexidine bathing for decreasing acquisition of MDROs and CRE (3). Immobilization leads to decubitis ulcers and failure to provide patient hygiene due to difficulties of bathing. Presence of decubitus ulcer and polymyxin, glycopeptide, and fluoroquinolone usage were found as independent risk factors for CRE colonization in our study. Correlatively, Swaminathan et al. reported that antimicrobial exposure is one of the main risk factors for CRE acquisition (10). 

Extended-spectrum beta-lactamase genes are most frequently isolated from *E. coli* and *K. pneumoniae* strains and the dominant genes are *CTX-M*, *SHV*, and *TEM* (21). Similarly, in the literature the most frequent CRE isolates are *K. pneumoniae*, *E. coli*, and *E. cloacae *(17,22). In a study conducted by Zarakolu et al. with a similar patient population as our facility, 276 CR *K. pneumoniae* isolates were investigated for carbapenemases, of which 270 produced OXA-48 type enzymes (22). Huang et al. investigated 323 OXA-48-producing Enterobacteriaceae isolates, of which 71.8% were defined as *K. pneumoniae*, 13.6% as *E. coli*, 7.4% as *E. cloacae*, 2.4% as *K. oxytoca*, and 2.4% as *Citrobacter freundii*. It has been reported that resistance to ertapenem is the most sensitive indicator of potential carbapenemase activity in CRE isolates that produce the OXA-48 type enzyme (23). In our study, carbapenem resistance was detected using conventional methods and semiautomated systems followed by E test confirmation. In the colonized patient group 75.5% *K. pneumoniae*, 15.5% *E. coli*, 4.2% *E. cloacae*, 1.4% *K. oxytoca*, and 1.4%* K. terrigena* were isolated from rectal swab cultures.

CRE-colonized patients had longer hospital stays before colonization and longer total hospital stays when compared with noncolonized patients. However, these factors were not statistically significant in randomized controlled studies (10,13). In our study, although the duration of total hospitalization was longer in the colonized group (P = 0.001), the time before colonization was not different. In accordance with the literature, there was no significant difference in mortality between the two groups in our study (13).

As a limitation, carbapenemase genes were not detected by PCR in this study. 

This is the first study determining the risk factors for rectal CRE colonization for adult patients in Turkey. Presence of decubitus ulcer and usage of polymyxin, glycopeptide, and fluoroquinolone were identified as risk factors for rectal CRE colonization. These results may provide guidance for outlining infection control programs to reduce CRE colonization in healthcare facilities in our region. According to these findings, appropriate interventions for CRE colonization should be emphasized with antibiotic stewardship and special attention should be given to antibiotic overuse. Furthermore, it must be noted that wound prevention and care are significantly important aspects of CRE colonization control.
